# Leukocytoclastic Vasculitis in a Patient with Ankylosing Spondylitis

**DOI:** 10.1155/2014/653837

**Published:** 2014-02-05

**Authors:** Senol Kobak, Hatice Yilmaz, Ahmet Karaarslan, Murat Yalcin

**Affiliations:** ^1^Department of Rheumatology, Faculty of Medicine, Sifa University, Bornova, 35100 Izmir, Turkey; ^2^Department of Orthopedic Surgery, Faculty of Medicine, Sifa University, Bornova, 35100 Izmir, Turkey

## Abstract

A 26-year-old male patient presented to our rheumatology clinic with pain, swelling and limitation of movement in his right ankle, and also purpuric skin lesions in the lower extremity pretibial region. He was asked questions, and he said that he had been having chronic low back pain and morning stiffness for the last few years. His physical examination revealed that he had arthritis in his right ankle, purpuric skin lesions in pretibial regions of both legs, and bilateral FABERE/FADIR positivity. The sacroiliac joint imaging and MRI revealed bilateral sacroiliitis findings, and the lateral heel imaging revealed enthesitis. HLA-B27 was positive. Skin biopsy from lower skin lesions was reported to be consistent with leukocytoclastic vasculitis. Based on clinical, laboratory, radiological, and pathological examinations, the patient was diagnosed with ankylosing spondylitis and leukocytoclastic vasculitis. Administration of corticosteroid, salazopyrin, and nonsteroid anti-inflammatory medications was started. Notable clinical and laboratory regression was observed during his checks 3 months later.

## 1. Introduction

Ankylosing spondylitis (AS) is a chronic inflammatory disease, which can involve the spine and the sacroiliac joint. Its most common symptoms are chronic low back pain and morning stiffness. Leukocytoclastic vasculitis (LV) is a disease characterized by inflammation of small veins [1]. Its etiology includes various causes such as drugs, chemicals, infections, malignancies, lymphoproliferative disorders, connective tissue diseases, and systemic inflammatory diseases [2, 3]. However, the cause cannot be detected in some of the cases, and these cases are considered idiopathic cases [4, 5]. In practice, LV is a pathology commonly seen in connective tissue diseases and/or vasculitides. Studies have shown that there is a relation between WG, PAN, sarcoidosis and rheumatoid arthritis, and LV [6–8]. Blanco et al. have also shown that collagen tissue diseases are the most common causes of etiology of leukocytoclastic vasculitis [9]. The primary event in formation of pathogenesis of LV is formation of immune complex on the vein wall. The process, which starts when immune complexes activate the complement system, results in the damaging of vein walls by inflammatory cells [10]. Coexistence of ankylosing spondylitis and leukocytoclastic vasculitis is very rare and is limited to only a few case reports. In this document, we report a case of AS coexisting with leukocytoclastic vasculitis.

## 2. Case Report

**Figure 1:**
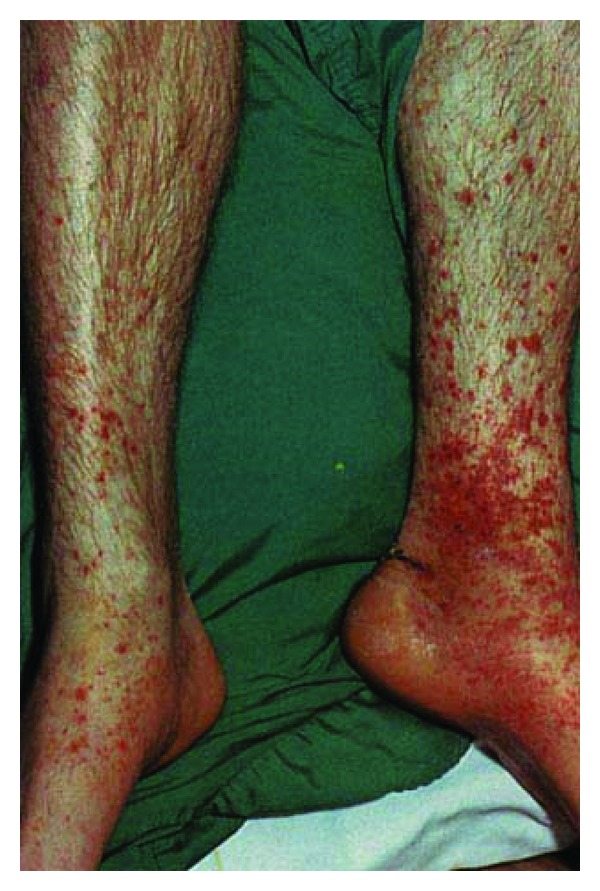
Purpuric skin lesions in the lower extremity pretibial region.

**Figure 2:**
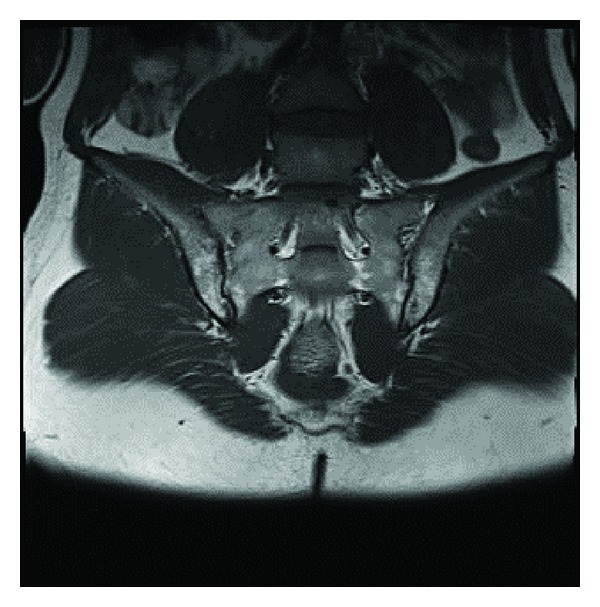
MRI of the sacroiliac joints revealed bilateral sacroiliitis.

A 26-year-old male patient presented to our rheumatology clinic approximately two weeks ago with pain, swelling and limitation of movement in his right ankle, and also purpuric skin lesions in the lower extremity pretibial region (Figure 1). He was asked questions, and he said that he had been having inflammatory low back pain and morning stiffness for the last few years. His other system questioning was normal. He had no history of medication use or past infection. His physical examination revealed that he had arthritis in his right ankle, purpuric skin lesions in pretibial regions of both legs, and bilateral FABERE/FADIR positivity. His laboratory findings were as follows: white blood cell: 11000 *μ*L, platelet: 476000 *μ*L, hemoglobin: 13.8 g/dL, hematocrit: 38.8%, SGOT: 37 U/L (0–40), SGPT: 39 U/L (0–41), blood sugar: 96 mg/dL (70–110), urea: 22 mg/dL (0–49), creatinine: 0.79 mg/dL (0.7–1.2), total protein: 7.4 g/dL (6.4–8.3), and serum albumin: 3.8 g/dL (3.4–4.8). His acute phase responses were as follows: C-reactive protein (CRP): 8.48 mg/dL (0–0.5) and erythrocyte sedimentation rate (ESR): 44 mm/h (0–15). His serological test results were as follows: rheumatoid factor (RF): negative, antinuclear antibody (ANA): negative, anti-CCP: negative, ANCA: negative, cryoglobulin: negative, complements (C3, C4): normal, and IgA: 508 mg/dL (90–453). His hepatitis serology (HbsAg, anti-HCV) and HIV were negative, and his thyroid function tests were found to be normal. His abdominal and lung images were normal. The sacroiliac joints imaging and MRI revealed bilateral sacroiliitis (Figure 2), and the lateral heel imaging revealed enthesitis. HLAB27 was positive. His measurements were as follows: BASDAI: 7 cm, BASFI: 5 cm, Schöber test: 4 cm, chest expansion: 4 cm, and finger to floor distance: 12 cm. Skin biopsy from lower skin lesions was reported to be consistent with leukocytoclastic vasculitis. Based on clinical, laboratory, radiological, and pathological examinations, the patient was diagnosed with ankylosing spondylitis and leukocytoclastic vasculitis. Administration of corticosteroid 16 mg/day, salazopyrin 2 g/day, and NSAII was started. Notable clinical and laboratory regression was observed during his checks 3 months later. It was observed that the arthritis in his right ankle regressed, and his skin lesions completely disappeared, and his low back pain and morning stiffness complaints also regressed. His check laboratory test results were ESR: 2 mm/h and CRP: 0.12 mg/dL. Outpatient polyclinic follow-up of the patient continues.

## 3. Discussion

Leukocytoclastic vasculitis was first defined in 1950s by Zeek as vasculitis in postcapillary venules after drug intake and was named “hypersensitivity vasculitis.” In the classification by Chapel Hill Consensus Conference in 1994, leukocytoclastic vasculitis was included in the “small vein vasculitis” based on the vein diameter involved [11]. Some authors state that cutaneous leukocytoclastic vasculitis is vasculitis limited to the skin, and some state that systemic involvement can be mild to moderate. While leukocytoclastic vasculitis is usually idiopathic, many factors are responsible for its etiology [12]. In all cases, which suggest small vein vasculitis, and especially in vasculitis cases limited to skin not accompanied by systemic findings, drug etiology must be considered. The drugs which cause leukocytoclastic vasculitis include penicillins, sulfonamides, NSAIIs, thiazides, retinoids, and antibiotics of the quinolone group. To consider that the vasculitis manifestation is due to a drug, rash must have occurred a short period after starting taking the drug, and the rash must have recessed when the drug is stopped, and there must have been cases reported with suspected drug. In our case, history and physical examination were used to investigate the relation between vasculitic rash, and this factor was ruled out. Viral hepatitis, HIV, and bacterial and fungal infections are included in the etiological infections. In our case, infections were ruled out with clinical, laboratory, and radiological imaging methods. In patients diagnosed with leukocytoclastic vasculitis, the tests primarily required are infectious serology, whole blood count, whole urine analysis, erythrocytes sedimentation rate, and liver and kidney function tests as well as lung imaging and skin biopsy. Although elevated erythrocyte sedimentation rate can be seen in more than 50% of the patients, apparent elevation as in our patient (>20–60 mm/hour) can be a sign of systemic disease. In the study by Sais et al., it has been found that elevated levels of leukocytosis, thrombocytosis, and erythrocyte sedimentation rate are correlated to systemic involvement [3].

Coexistence of ankylosing spondylitis and leukocytoclastic vasculitis is very rare and is limited to only a few case reports. Hsu et al. have reported IgA nephropathy and leukocytoclastic vasculitis accompanying ankylosing spondylitis in a patient [13]. IgA and C3 accumulation were shown in kidney and skin biopsies and were considered to have a role in the pathogenesis of ankylosing spondylitis [14]. Similarly, inflammatory bowel disease and IgA-associated glomerulonephritis and cutaneous vasculitis case coexisting with inflammatory bowel disease and ankylosing spondylitis were reported in this study [14]. The IgA level of our patient was also high. Although, in many studies, the serum IgA levels were found to be high in patients with AS, it is controversial whether the IgA levels are an indicator of disease activity. Literature searches have shown that there have been case reports with leukocytoclastic vasculitis accompanied by IgA nephropathy [13, 14]. This suggests that the IgA level can be responsible for etiology in the development of leukocytoclastic vasculitis in cases with AS.

As a result, the fact that our patient did not have a history of drug use and other possible etiological causes such as infections that were ruled out, that sedimentation was clearly high, that the patient had leukocytosis and thrombocytosis, and that he had arthritis in his right ankle primarily suggested systemic disease as per the literature data. The SIJ MRI has revealed bilateral sacroiliitis, and lateral heel imaging has revealed enthesitis and HLAB27-positivity, and consistency of the skin biopsy from skin lesions has revealed coexistence of leukocytoclastic vasculitis and ankylosing spondylitis.

According to the cases reported where ankylosing spondylitis and leukocytoclastic vasculitis coexist in the literature, ankylosing spondylitis can be one of the causes of leukocytoclastic vasculitis. Considering that the actual important point in the treatment of leukocytoclastic vasculitis is treatment of the cause, it is one of the diseases that need to be taken into account in discriminative diagnosis.
